# Notes of a Recent Visit to Some of the American Asylums

**Published:** 1851-07-01

**Authors:** J. Ball


### NOTES OF A RECENT VISIT TO SOME OF THE AMERICAN ASYLUMS.

BY J. BALL, ESQ., M.R.C.S.E.

The physical aspect of a country has, we believe, an influence upon the character of its inhabitants; and the grandeur of American scenery—its extensive forests—its vast lakes—and its magnificent rivers and waterfalls—are morally suggestive of great undertakings. Hence the social institutions of America are for the most part conceived upon a large and extensive scale. Their public buildings, their street architecture, the style of their principal cities, have a noble character, breathing rather an air of ostentatious monarchy than of simple republicanism. The progress of the Americans in science and literature has been signal; and in that department of the profession to which this journal is dedicated, they have evinced an energy worthy of a free and enlightened people. At this moment there are philosophical works publishing in America that would do honour to Great Britain. And to what is this to be attributed but to that spirit of liberty which emancipates their philosophy from scholastic chains, which in European universities too often restrict the independence of the inquiring mind. Liberty and philosophy must ever go hand-in-hand; without intellectual liberty there can be no progression of thought—no positive advancement of knowledge. The liberal scale upon which all the great institutions of America are founded, seems to outstrip European competition. Their hospitals for the sick are admirably designed and organized—nay, we believe that, in many respects, they are even better managed than many in this country. Their architectural designs are vast and comprehensive. Their wards, day-rooms, sleeping-rooms, baths, kitchens, &c., are lofty and spacious, and well arranged; and so, likewise, we gather from the various reports which we have from time to time analyzed, that their lunatic asylums are admirably constructed, and managed upon the best and most enlightened principles.

Having paid recently a visit to the United States, and being much interested in the subject of insanity, we inspected some of the principal asylums in that country, a short account of which we purpose now giving. Arriving in the state of New York, we found nearly in its centre the city of Utica, and about a mile and a half distant from it is the New York State Lunatic Asylum. The main building, we may observe, is constructed of hewn limestone; it has two wings, is three stories in height, and occupies an area of 550 feet. The centre of the main building is appropriated to the offices and private apartments belonging to the officers of the establishment, and there is accommodation for 600 patients. The halls are 225 feet in length, and 13 feet in width, with sleeping apartments on either side, as well as a sitting-room, dining-room, and bathing-room, *en suite*, capable of accommodating 30 or 40 patients. There are as many as 380 single rooms for patients, 24 for attendants, and 20 associated dormitories, each of which will accommodate from 5 to 12 patients. There are besides two large rooms fitted up as hospitals, and a chapel which will accommodate a congregation of 500 persons. In addition to which there are various shops—1 plumber's, 2 joiners', 1 tailor's, and two printing-shops, and several work-rooms for females. It is lighted by gas, warmed by hot air, conducted by flues from the basement, and there is a plentiful supply of water derived from a canal half a mile from the building. The management of the asylum is vested in a board of nine directors, who are appointed by the legislature; the majority of whom are required to reside within five miles of the institution. They hold office for three years, but may within that period at any time be removed by the senate of the legislature. This board appoints the superintendent and treasurer, and is empowered to enact such bye-laws as may be deemed expedient for arming the other officers with authority; it also determines the conditions for the admission and the support of patients, and the period of their discharge. Its staff of officers is far more effective than in any of our country asylums; it consists of a resident superintendent and physician, and three assistant-physicians; a steward, matron, and two apothecaries. The power of the superintendent is absolute. He has the entire medical and moral control of the establishment, the other officers acting under his directions. His income is 2000 dollars per year, with board and residence; and each assistant-physician has 600 dollars per year, also with board and residence.

The following table exhibits the number of patients in the asylum during the year 1849:—

| | Males. | Females. | Total. |
|---|---|---|---|
| Number of patients, January 1, 1849 .......... | 241 | 254 | 495 |
| Admitted during the year ...................... | 192 | 170 | 362 |
| Total number in the course of the year ........ | 433 | 424 | 857 |
| Discharged recovered ...................... | 113 | 90 | 203 |
| „ improved ......................... | 37 | 29 | 66 |
| „ unimproved ....................... | 70 | 22 | 48 |
| Died ..................................... | 35 | 34 | 69 |

By this table it will be observed, that 857 cases were under treatment during the year; and of these, 203 recovered, giving a per centage of 56 on the admissions during the year, or 23½ on the whole number of patients in the asylum. The deaths were 69, or 8 per cent. on the patients for the year; and of these, 28 died from epidemic diseases, viz., small-pox and dysentery. This asylum escaped the cholera, although that disease was during the year fatal in the neighbourhood.

The following return shows the causes of the 69 deaths:—

DISEASES OF THE HEAD.

| | |
|---|---|
| Meningitis ........................................ | 7 |
| Epilepsy ........................................ | 3 |
| Apoplexy ........................................ | 1 |
| General paralysis ................................ | 4 |

DISEASES OF THE CHEST.

| | |
|---|---|
| Consumption ........................................ | 6 |
| Pneumonia ........................................ | 2 |
| Hydrothorax ........................................ | 1 |

DISEASES OF THE ABDOMEN.

| | |
|---|---|
| Dysentery | 14 |
| Diarrhœa | 3 |
| Perforation of intestine | 1 |

DISEASES OF UNCERTAIN SEAT.

| | |
|---|---|
| Small-pox | 14 |
| Exhaustion after excitement | 5 |
| Marasmus | 2 |
| General dropsy | 1 |
| Suicide | 1 |
| Puerperal fever | 1 |
| Spinal disease | 1 |
| Erysipelas | 1 |
| Old age | 1 |

There being 14 deaths by small-pox is somewhat remarkable, as it is difficult to trace the origin of the disease. The first occurred in a patient who had been seven months in the asylum; no case of small-pox had been in the neighbourhood, nor had any patient, as far as could be ascertained, come from a part in which that disease prevailed. The 14 deaths from dysentery were, with two exceptions, all demented and incurable.

The following return shows the whole number of patients admitted, recovered, and the deaths which have occurred since the opening of the asylum in 1843.

| | |
|---|---|
| Number of patients admitted | 2376 |
| Recovered | 1017 |
| Improved | 419 |
| Not improved | 222 |
| Died | 269 |
| Remaining in the asylum | 449 |

Hence, 43 per cent. recovered, 18 per cent. improved, and the deaths were $11\frac{1}{2}$ per cent.

In this, and all other state government asylums throughout the United States, private patients are received at a charge above that of pauper patients—the latter are admitted at two dollars per week, the former pay from two dollars and fifty cents to four dollars. No distinction is made between these two classes of patients—they all live in the same halls and dine at the same tables. This plan appears to answer well in America, where there is not the distinction between classes of society which prevails in England, and where every person can, at the expense of the state, receive at the public schools a liberal education.

The classification of patients according to their mental state is very perfect in this asylum, there being no less than twelve different classes, the benefit derived from which arrangement is obvious. The wards may be observed to be quiet, and it very rarely happens that any patient is placed in seclusion or under restraint, which, when we consider the large number of recent cases admitted, is highly creditable to the institution, and sufficiently evinces the able manner in which it is conducted by its present experienced superintendent, Dr. Benedict, who has only recently been appointed to this office, vacated by the death of the late Dr. Brigham, whose psychological investigations we have frequently had occasion to notice. He was an accomplished physician, and an amiable and good man.

The occupations and amusements provided for the patients constitute one of the most prominent and praiseworthy features of this asylum.

Besides the workshops we have mentioned, there is a farm of 130 acres, at which 512 patients might be observed to be farmers interested in their agricultural pursuits, and 151 labourers. The employment is much liked, and the patients who are capable, are very ready and willing to be so occupied. The women, during the day, ride, walk, and employ themselves in making clothes, and various kinds of needle and fancy work. The evenings are invariably devoted to some kind of rational amusement; such as lectures, reading, music, dancing, chess, tableaux, &c. Frequently during the winter, dramatic representations are got up, the characters being performed entirely by the patients. There is also a school in the asylum, daily open for persons who are disposed to receive instruction.

In one of the halls the patients hold an annual fair, at which a vast variety of fancy articles, made by the patients themselves, are exhibited and sold—such as carvings in wood, ivory, bone, needle-work, netting, &c. The proceeds arising from these fairs in five years yielded 1000 dollars, with which an organ for the chapel was bought, as well as musical instruments and books for a brass band. It remains only to add, that a clergyman is attached to the institution, and there is generally a large attendance of patients in the chapel every Sunday. The management of this asylum reflects the greatest credit upon the resident superintendent, the physician, and assistant-physicians, and indeed upon all the officers connected with it. It is by their united zeal and their conjoint labours that results so satisfactory are produced. Persons unacquainted practically with the management of a lunatic asylum, and who do not know how much can really be effected to instruct, improve, occupy, amuse, and ameliorate generally the condition of the insane, may suspect that such accounts as the above are exaggerated—that there is an air of Utopianism about them which cannot be realized—but our own experience—had we not even crossed the Atlantic—is otherwise, and the greatest encouragement we have derived in the practice of this anxious branch of our profession, has been the success which has attended upon a large scale, the well considered and judicious management of both public and private asylums.

Let us, however, now leave the New York State Lunatic Asylum, and while upon our arrival trip, visit another of these admirable institutions, the Bloomingdale Asylum—the history and statistics connected with which, we noticed at some length in the second volume of this journal.* This asylum is delightfully situated, about four miles from New York, a quarter of a mile, or thereabouts, from Hudson's River, and commands a magnificent prospect over the surrounding country. When this establishment was opened there were in the United States only four other institutions exclusively devoted to the reception and treatment of the insane. The building is constructed of brown freestone; and in its rear are two detached lodges for the more violent and noisy patients. The interior architectural arrangements, considering the premises were built thirty years ago, are excellent; the corridors wide and lofty, with sleeping apartments opening into them on either side, capable of holding from one to four patients. But the majority of these are single rooms. In each hall there is a dining-room, sitting-room, bath-room, *en suite,* and the apartments are for the most part heated as at the New York State Asylum, by hot air sent from furnaces on the basement story. Each sitting-room, however, has an open coal fire during winter, which, if less economical and convenient in some respects, is certainly more cheerful, which is a more important consideration than might cursorily be supposed. The apartments in which patients are assembled heated by any other con-

* "The Journal of Psychological Medicine and Mental Pathology," vol. 2, p. 189, 1849.

trivance than an open fire-grate, must be, morning, noon, and evening, during the dreary days of winter, miserably gloomy. The poor insane, conscious many of them that their malady has imposed upon them a species of temporary imprisonment, are very sensible of all external impressions; they should be surrounded with everything that has a cheerful aspect; the very colour of the walls of the apartments has an effect upon their mind. To return. The airing courts are spacious, and tastefully planted with trees, among which seats are distributed, and on the men's side is a "ten pin alley," a favourite game in America, which affords healthy exercise as well as recreation for patients. The whole establishment is well supplied with water derived from the Croton Water Works, the source of the city supply.

The internal government of this institution—which is a branch of the New York State Asylum—is vested in a committee of six officers, who are appointed by the board of governors of the general hospital, from among their own numbers; the proceedings of this committee being subordinate to those of the general board. The resident officers are the physician, warder, matron, and apothecary. There is no assistant physician, as in nearly all the other American asylums. The following table shows the number of admissions, discharges, and deaths during the year 1849:—

| | Males. | Females. | Total. |
|---|---|---|---|
| Number of patients in the house, January 1, 1849.. | 59 | 60 | 119 |
| Number admitted during the year .............. | 58 | 37 | 95 |
| Whole number of cases in the asylum .. | 117 | 97 | 214 |
| Discharged during the year, recovered .......... | 26 | 18 | 44 |
| " " improved .......... | 17 | 16 | 33 |
| " " not improved........ | 11 | 2 | 13 |
| Died .................................... | 13 | 8 | 21 |

Ten of the above cases were from intemperance; eight having been admitted during the year, and two old cases being already in the asylum. Seven of the recoveries were of this description, leaving only thirty-seven cases of recovery from insanity proper. Of these the following returns, showing the duration of the attack, may be read with interest:—

| | | | | | | |
|---|---|---|---|---|---|---|
| 27, | at the time of admission, | had | been | insane less than | 6 months | |
| 7 | " | " | " | " | 1 year | |
| 3 | " | " | " | " | 2 years | |
| 1 | " | " | " | " | 1 month | |
| 5 | " | " | " | between | 1 and 2 months | |
| 4 | " | " | " | " | 2 and 3 | " |
| 8 | " | " | " | " | 3 and 4 | " |
| 7 | " | " | " | " | 4 and 5 | " |
| 1 | " | " | " | " | 5 and 6 | " |
| 6 | " | " | " | " | 6 and 9 | " |
| 2 | " | " | " | " | 9 and 12 | " |
| 2 | " | " | " | " | 12 and 15 | " |
| 1 | " | " | " | " | 35 and 36 | " |

We observe by this return, the larger number of recoveries took in those who were admitted into the asylum while the attack was yet recent, and evinces, as so frequently has been proved, the expediency in all cases of insanity, of early treatment. The present talented physician of this asylum, Dr. Nicholls, has frequently occasion to lament the premature removal of a large number of patients, owing to their friends being in narrow circumstances and unable to continue their support in the asylum. The number of cures is hereby greatly reduced; and it is observed that those who are thus prematurely discharged often become absolutely incurable. The mistaken kindness of relations and friends who are apt to listen to the entreaties of persons who are only partially recovered, leads to the same unhappy result; prematurely discharged, such patients when all moral restraint is removed, speedily relapse, and return either to this, or are sent to some other asylum, with the chances of recovery considerably diminished. Such cases are marked as having been discharged improved.

The deaths were twenty-one, or 9¾ per cent. for the whole number under treatment during the year, and the causes of death in these cases were as follows:—

| | |
|---|---|
| Apoplexy | 4 |
| Typo-maniacal delirium | 2 |
| Delirium tremens | 1 |
| Dysentery | 3 |
| Diarrhœa | 4 |
| Cancer | 1 |
| Suicide | 2 |
| Cause not stated in the report | 4 |

This asylum escaped the cholera, which was prevalent in the neighbourhood, the city of New York having suffered greatly from the ravages of this pestilence.

Only private patients are now admitted into this asylum; formerly pauper lunatics were received, but these have been transferred to the city Pauper Asylum, on Blackwell's Island, and the State Asylum, at Utica. The charge for private patients varies from four to fifteen dollars per week. The amusements consist in lectures, evening parties, occasional balls; which are always conducted with great spirit and decorum. The majority of patients belonging to the upper and middle classes are not accustomed to bodily labour, therefore few are willing to work in the farm or gardens; the former consists of fifty acres, the latter are large and well laid out, with an excellent conservatory. A carriage and horses are kept for the use of the patients. Restraint is occasionally, but very rarely, had recourse to, and in such cases Wyman's bed-strap is the apparatus used, which has simply the effect of keeping the patient in bed and preventing violence to others.

Bidding adieu to the excellent and well-conducted asylum, let us direct our course towards Blackwell's Island, one of those small *oases* with which Long Island Sound abounds. Here we find a pauper asylum, erected by the corporation of New York, for patients belonging to that city, which was opened in the year 1839. The island upon which it is situated is five miles from the city hall, and has the advantage, from its insular position, of being isolated yet of easy access. The building is constructed of stone, and consists of two wings radiating from a centre; which central portion is appropriated to offices and apartments for the resident staff. There are also two detached buildings, or lodges, as they are termed, for refractory and dirty patients. The following table exhibits the number of admissions, discharges, and deaths, during the year 1849.

| | Males. | Females. | Total. |
|---|---|---|---|
| Number of patients, January 1, 1849 | 187 | 250 | 437 |
| Admitted during the year | 229 | 230 | 459 |
| Discharged | 145 | 138 | 283 |
| Died | 85 | 127 | 212 |
| Remaining in the asylum, January 1, 1850 | 181 | 215 | 401 |

The number of deaths here is very large, nearly 24 per cent.; but 86 of these died from Asiatic cholera, and 48 from diarrhœa, showing how severely this asylum suffered from these diseases. Those among whom the greatest fatality occurred were demented and of dirty habits. The largest number of deaths occurred in the months of June and July; especially on the 9th and 16th of July, the preceding days having been exceedingly damp and unpleasant. Eight per cent. of those who died had been in the asylum more than ten years, and probably more than one-half had been insane from ten to twenty-five years. The subjoined table shows the causes of death in 212 cases:—

| | |
|---|---|
| DISEASES OF THE BRAIN. | |
| Congestion of the brain | 12 |
| Apoplexy | 5 |
| Epilepsy | 5 |
| Paralysis | 2 |
| General paralysis | 3 |
| Delirium tremens | 3 |
| Softening of the brain | 1 |
| DISEASES OF THE CHEST. | |
| Phthisis | 21 |
| Typhoid pneumonia | 1 |
| DISEASES OF THE ABDOMEN. | |
| Asiatic cholera | 86 |
| Diarrhœa | 48 |
| Dysentery | 4 |
| Albuminuria | 1 |
| Chronic peritonitis | 1 |
| SEAT OF DISEASE UNCERTAIN. | |
| Scrofula | 1 |
| Scurvy | 1 |
| Suicide | 1 |
| Typhoid fever | 8 |
| Erysipelas | 2 |
| Dropsy | 1 |
| Old age | 4 |
| Exhaustion from exposure to cold before admission | 1 |
| Total deaths from all causes | 212 |

Of the 283 patients discharged, 212 had recovered, and 60 were sufficiently improved to return to their friends; thus giving nearly 50 per cent. of recoveries on the admissions, and a little under 25 per cent. on the whole number under treatment during the year.

The following table shows the form of the disease in the 283 patients discharged during the year:—

| | Recovered. | Improved. | Not improved. | Total. |
|---|---|---|---|---|
| Mania | 132 | 31 | .. | 163 |
| Mania, partial | 30 | 16 | .. | 46 |
| Mania, puerperal | 8 | .. | .. | 8 |
| Mania, moral | 1 | 1 | 1 | 3 |
| Dementia | 1 | 12 | 3 | 16 |
| Dementia senile | .. | .. | 3 | 3 |
| Delirium tremens | 36 | .. | .. | 36 |
| Hysteria | 1 | .. | .. | 1 |
| Febrile delirium | 3 | .. | .. | 3 |
| Improper subjects for the asylum | .. | .. | 4 | 4 |

By this table it will be perceived that 40 of the cases of recoveries were not, strictly speaking, cases of insanity, which, being deducted, leaves only 37½ per cent. of recoveries on the number of admissions of insanity proper.

The following is the term of residence of those discharged:—

| | Recovered. | Improved. | Not improved. | Total. |
|---|---|---|---|---|
| Less than 3 months | 130 | 34 | 5 | 169 |
| From 3 to 6 months | 47 | 10 | 4 | 61 |
| From 6 to 12 months | 23 | 7 | 0 | 30 |
| From 1 to 3 years | 11 | 6 | 2 | 19 |
| From 3 to 6 years | 1 | 3 | 0 | 4 |

The medical staff of this asylum is composed of two visiting physicians —Drs. Ogden and Williams; a resident physician and superintendent—Dr. Roxney and two assistant physicians, the three latter gentlemen being resident officers. Originally, four visiting physicians were appointed; the other two were Drs. Pliny Earle and Macdonald. The former was prevented from acting by being appointed physician to the Bloomingdale Asylum. The latter gentleman died in May, 1850. These vacancies have not been filled up, and the duties, therefore, of visiting physicians, devolve entirely on Drs. Ogden and Williams. It should be mentioned, that there is a large library in this asylum, with a good selection of works on history, biography, and general literature, to which the patients at all hours of the day have free access.

Having completed our survey of Blackwell Island, we had some idea of winging our way over to the Ohio Lunatic Asylum, and the Pennsylvania Hospital for the insane, and afterwards visiting the establishment of idiots at the Massachusetts; but the weather was cold and stormy; pressing engagements awaited us at home; so, thanking our industrious and talented guide for his friendly services, we steered southward, promising him that we should avail ourselves of his information in the present number of *The Journal of Psychological Medicine.*

